# Editorial: Therapeutic Modulation of the Complement System: Clinical Indications and Emerging Drug Leads

**DOI:** 10.3389/fimmu.2019.03029

**Published:** 2020-01-09

**Authors:** Dimitrios C. Mastellos, Edimara S. Reis, John D. Lambris

**Affiliations:** ^1^National Center for Scientific Research “Demokritos”, Athens, Greece; ^2^Department of Pathology and Laboratory Medicine, Perelman School of Medicine, University of Pennsylvania, Philadelphia, PA, United States

**Keywords:** complement system, clinical trials, therapeutics, diseases, drug candidates

Over the last two decades, our perception of the complement system as an innate immune sentinel that is solely responsible for pathogen elimination has been fundamentally transformed to that of a multi-tasking immune system that intricately coordinates both innate and adaptive immune responses (1). This is achieved through extensive crosstalk of complement effectors with multiple pattern recognition and proinflammatory signaling systems both in the intravascular space and in intracellular compartments. Complement is considered a fundamental humoral branch of innate immunity that rapidly responds to danger signals coordinating with other key defense systems, such as the endothelial barrier, contact activation, and coagulation systems (2). Through these reciprocal interactions, complement contributes to the maintenance of host immunosurveillance and tissue homeostasis. Its rapid and forceful activation in the bloodstream not only ensures the effective containment of microbial infections through opsonophagocytic mechanisms, but also alerts the adaptive immune compartment to ensure the mounting of a proper humoral response against foreign antigens. However, there is a lurking “dark side” that can lead complement astray, fueling a self-perpetuating vicious cycle of inflammation that results in persistent immune activation and irreversible tissue injury in both acute and chronic pathologies (2). Indeed, complement dysregulation or excessive activation have been recognized as key pathogenic drivers in a wide spectrum of inflammatory, immune-mediated, and age-related neurodegenerative diseases (3).

More than a decade after the clinical approval of the first complement-specific drug, the C5-targeting monoclonal antibody eculizumab (Soliris, Alexion), the complement drug space is ripe with new opportunities for therapeutic intervention at multiple steps of the cascade (4). The clinical success of complement-based therapy has been further consolidated through the recent approval of eculizumab in two indications of the neurological spectrum: generalized myasthenia gravis (gMG) and neuromyelitis optica spectrum disorder (NMOSD) (3). Furthermore, several drug candidates acting upstream of C5, or on downstream effectors, have advanced to Phase III trials in renal, hemolytic and ocular indications, promising broader or more tailored clinical benefit than anti-C5 (3). Taken together, these game-changing developments have laid the groundwork for advancing a new generation of complement therapeutics to the clinical stage, in a spectrum of indications ranging from ocular, neurodegenerative, and thromboinflammatory disorders, to cancer, periodontal diseases, chronic hemolytic anemias, ischemia-reperfusion organ injury, antibody-mediated transplant rejection, and hemodialysis-triggered inflammation (5–7).

In addition to this expanding clinical landscape, the growing commitment of the biopharmaceutical industry in the complement drug space is readily reflected in the recently announced multi-million acquisitions of complement-dedicated startups by global healthcare companies (8). These widely publicized and lucrative corporate decisions have not only bolstered confidence in the clinical potential of complement intervention but also raised awareness about regulatory issues pertinent to drug market competition, the prevalence of monopolizing practices in the complement drug space and the relationship between true patient benefit, optimal drug or target selection, and incurred patient costs.

This Research Topic attracted leading academic and clinical experts in complement pathobiology and clinical translation, providing a forum to critically discuss the latest developments in complement drug discovery, from a disease-oriented perspective. While the list of selected clinical indications is certainly not exhaustive, it does illustrate the diversity of therapeutic approaches currently adopted in the field. Our topic includes examples of transformative clinical research that may soon change the treatment landscape in several complement-mediated diseases, while challenges faced along the drug discovery path are also discussed. Overall, emphasis is placed on the potential of the drug development pipeline to deliver to the clinic new complement-targeted therapies tailored to specific diseases.

Hemolytic conditions fueled by complement dysregulation have long remained in the crosshairs of the biopharma industry. Complement dysregulation is recognized as the main pathogenic driver in paroxysmal nocturnal hemoglobinuria (PNH) and as a major exacerbator of autoimmune hemolytic anemias (i.e., cold agglutinin disease, CAD) (3). In fact, PNH has served as a model for benchmarking new complement therapeutics in the clinical setting. While anti-C5 therapy has transformed the clinical course of PNH abrogating intravascular hemolysis and lowering thrombotic risk, there is still an unmet clinical need with regard to residual anemia that is mainly attributed to extravascular C3-mediated hemolysis. In this topic, Risitano et al. provide an overview of the clinical programs targeting complement upstream of C5 and propose that proximal complement inhibition (at the level of C3 or AP convertase) may drastically improve the hematological response in PNH patients who respond insufficiently to anti-C5 agents. The clinical success of anti-C5 therapy in atypical hemolytic uremic syndrome (aHUS) has fueled discussions on the pathogenic involvement of complement dysregulation in a broad spectrum of thrombotic microangiopathies (TMAs). Gavriilaki et al. provide an overview of the clinical landscape and pathophysiological “conundrum” of complement-mediated TMAs and critically address the feasibility of complement inhibition in a spectrum of TMAs with a pathogenic involvement of complement.

Excessive complement activation has been linked to multiple pathological sequelae associated with traumatic or infectious insults leading to multiple organ dysfunction (4). In this topic, Karasu et al. discuss the broad spectrum of pathophysiological consequences of deregulated complement activation in polytrauma, hemorrhagic shock, and sepsis. They also review complement therapeutics which have shown promise as treatment options for these severely debilitating conditions.

Owing to its distinct anatomy and physiology, the kidney glomerulus is endowed with increased susceptibility to complement-mediated damage (9). Complement dysregulation underpins a wide range of glomerular pathologies and several complement therapeutics are currently in clinical development for renal indications. Zipfel et al. present an overview of the pathophysiological traits of several renal diseases linked to complement dysregulation, including aHUS, anti-neutrophil cytoplasmic antibody mediated vasculitis (ANCAV), C3 glomerulopathy, and IgA nephropathy. They also discuss ongoing clinical trials evaluating the efficacy of complement inhibitors in each of these renal indications. Remaining in the renal space but focusing more on the clinical promise of complement therapeutics in kidney transplantation and organ accommodation across HLA and ABO barriers, Tatapudi and Montgomery discuss the pivotal pathogenic role of complement effectors in acute antibody-mediated rejection (AMR) during renal transplantation. They provide a comprehensive evaluation of various anti-complement agents in ongoing clinical trials of kidney AMR, weigh in on the potential of complement modulation in highly sensitized renal graft recipients and discuss the prospects and challenges of complement-based intervention in xenotransplantation. From a different perspective, van Zanden et al. focus on the pathophysiology of early tissue damage in the deceased organ donor, attempting to bridge a knowledge gap about the role of complement effectors in this process. The authors discuss preclinical evidence indicating that complement inhibition in the donor might be a promising therapeutic strategy to improve the quality of various donor organs for transplantation.

The discovery of complement gene polymorphisms that significantly elevate the risk of developing age-related macular degeneration (AMD) has sparked a fertile investigation into potential therapeutic avenues for treating ocular inflammation in AMD (3). Here, Park et al. review the evidence linking AP dysregulation with retinal inflammation and photoreceptor loss in AMD, and critically discuss ongoing clinical programs focused on therapeutic targeting of C3, FD, properdin, C5, and MAC in patients with geographic atrophy, a dry form of advanced AMD for which there is currently no approved therapy.

The role of complement in the development of chronic neuroinflammatory disorders has been documented in elegant preclinical models (10). Moreover, the recent approval of anti-C5 therapy as a treatment option for NMOSD and gMG has rekindled the interest of big biopharma in developing complement therapeutics in the neurological space. In this topic, Carpanini et al. discuss the challenges and opportunities of targeted complement modulation in acute or chronic CNS disorders and explore ways to improve drug access into the brain and tailor anti-complement therapies for CNS diseases. Focusing on complement intervention in acute ischemic stroke, Clarke et al. review the impact of complement inhibition on cerebral tissue injury and repair following ischemic damage. Importantly, they argue that successful clinical translation of complement therapies in stroke patients will depend on selection of the right therapeutic window, with early intervention during the ischemia-reperfusion phase favored over delayed intervention during cerebral tissue repair.

Complement acts as a “double-edged sword” in cancer immunity, potentiating antibody-mediated tumor cytolysis, but also promoting tumor-associated inflammation and immunosuppression in the tumor microenvironment (5, 11). The protumorigenic activities of complement have attracted considerable attention in recent years and complement therapeutics have recently entered clinical development as modalities for boosting the anti-tumor efficacy of immune checkpoint inhibitors. In this regard, Pio et al. provide a comprehensive overview of the central role of complement in the cancer-immunity cycle and discuss the emerging prospects of introducing complement modulation in combination immunotherapies for various cancer types. Besides cancer, complement activation has long been implicated in the pathogenesis of autoimmune diseases but clinical translation in this area has remained a conundrum. In this Topic, Thurman and Yapa discuss the evidence linking complement deregulation to autoimmune pathologies and evaluate the feasibility of complement inhibition in the clinical setting. Periodontal disease is fueled by a dysbiotic oral microbiota that thrives on oral inflammation and leads to the destruction of tooth supporting tissues. Hajishengallis et al. summarize the body of evidence supporting the pathogenic involvement of complement in oral inflammation, discuss the therapeutic efficacy of C3-targeted intervention in primate models of natural or induced periodontal disease and provide a robust rationale for ongoing clinical trials of C3 inhibitors in human periodontitis.

With a growing interest in clinical complement intervention and an expanding list of ongoing clinical trials, the need for robust patient stratification ahead of clinical trials is accentuated and the use of sensitive diagnostic tools and bioassays for monitoring complement activation in patients receiving anti-complement therapy has become a priority. Mohebnasab et al. address this priority by providing an overview of the appropriate technology platforms for complement biomonitoring and assess the challenges for reliable complement diagnostics in a number of clinical disorders.

Hardly ever before has the complement “space” witnessed such a vibrant and transformative set of defining developments in all fronts, as in recent years. Indeed, molecular and structural insights have revealed new functions and mechanisms of this innate immune system, while more refined animal disease models, high-throughput analytical tools, and high-dimensional technologies are now illuminating new pathogenic pathways in a tissue-specific manner and at unprecedented single-cell resolution. More importantly, human clinical studies have now begun to unveil significant causative associations between distinct complement pathways and processes underlying disease progression or therapy response. Encouraging results from ongoing clinical trials with complement inhibitors are paving the way for new and more efficacious therapies in both rare and more common clinical indications.

It is our conviction that this timely collection of review articles faithfully illustrates both the breadth and scope of research innovation, elegant therapeutic drug design and clinical translational effort invested in the complement drug space.

## Author Contributions

DM drafted the manuscript. ER and JL reviewed the manuscript and provided critical revisions. All authors contributed to the conception and design of this work, read the final version, and approved the submitted manuscript.

### Conflict of Interest

JL is the founder of Amyndas Pharmaceuticals, which is developing complement inhibitors for therapeutic purposes. JL is the inventor of patents or patent applications that describe the use of complement inhibitors for therapeutic purposes, some of which are developed by Amyndas Pharmaceuticals. JL is also the inventor of the compstatin technology licensed to Apellis Pharmaceuticals [i.e., 4(1MeW)7W/POT-4/APL-1 and PEGylated derivatives such as APL-2/pegcetacoplan]. The remaining authors declare that the research was conducted in the absence of any commercial or financial relationships that could be construed as a potential conflict of interest.
